# Influence of Pozzolanic Additives on the Structure and Properties of Ultra-High-Performance Concrete

**DOI:** 10.3390/ma18061304

**Published:** 2025-03-16

**Authors:** Jurgita Malaiškienė, Ronaldas Jakubovskis

**Affiliations:** 1 Laboratory of Composite Materials, Institute of Building Materials, Faculty of Civil Engineering, Vilnius Gediminas Technical University, Sauletekio av. 11, 10223 Vilnius, Lithuania; 2 Laboratory of Innovative Building Structures, Faculty of Civil Engineering, Vilnius Gediminas Technical University, Sauletekio av. 11, 10223 Vilnius, Lithuania; ronaldas.jakubovskis@vilniustech.lt

**Keywords:** high-performance concrete, pozzolanic additives, mechanical and durability properties, cement hydration

## Abstract

The aim of this paper is to analyse the influence of the following different supplementary cementitious materials (SCMs): milled quartz sand, microsilica, waste metakaolin, milled window glass, and a binary additive made of one part waste metakaolin and one part microsilica, on the properties of ultra-high-performance concrete, and choose the best additive according to the physical, mechanical, and structural properties of concrete. In all mixes except the control mix, 10% of the cement was replaced with pozzolanic additives, and the changes in the physical, mechanical, and structural properties of the concrete were analysed (density, compressive strength, water absorption, capillary water absorption, degree of structural inhomogeneity, porosity, freeze–thaw resistance prediction coefficient Kf values); X-ray diffraction analysis (XRD) and scanning electron microscopy analysis (SEM) results were then interpreted. Concrete with microsilica and the binary additive (microsilica + metakaolin) was found to have the highest compressive strength, density, closed porosity, and structural homogeneity. Compared to the control sample, these compositions have 50% lower open porosity and 24% higher closed porosity, resulting from the effect of pozzolanic additives, with which the highest density and structural homogeneity was achieved due to the different particle sizes of the additives used.

## 1. Introduction

Advancements in concrete technology have allowed the creation of concrete mixtures with compressive strengths, toughness, and ductility close to those of metallic materials [1]. Concretes with compressive strengths greater than 120 MPa are commonly called ultra-high-performance concrete (UHPC) [2]; high-performance concrete usually refers to concrete with compressive strength after 28 days above 70–80 MPa. In addition to high compressive strength, UHPC features outstanding durability properties with regard to water permeability, freeze–thaw resistance and carbonation rates almost ten times lower compared to conventional concrete [3,4]. Both the extreme compressive strength and enhanced durability properties of UHPC result from high particle packing density and low capillary porosity [5]. This makes UHPC applicable for slender and complex structural elements, bridge girders, or thin cladding panels [6]. Nevertheless, practical applications of UHPC are still scarce, mostly due to the relatively high cost of materials. The price of commercially available UHPC mixes depends on the country, and ingredients can go up to 1500 EUR/m^3^, compared to ordinary concrete at 50 to 200 EUR/m^3^ [7].

In addition to the high initial cost, UHPC mixes also have adverse environmental impacts due to the enormous carbon footprint of their raw materials. The embedded CO_2_ emission of UHPC mixes ranges between 600 and 1200 kg/m^3^. This emission rate is mostly caused by the high amount of cement (700–1200 kg/m^3^) and silica fume (100–500 kg/m^3^) in the composition of UHPC [8,9]. In addition, natural sand and coarse aggregate (priced around 15 EUR/m^3^) are generally replaced with finely ground quartz sand or quartz powder (priced around 150 EUR/m^3^), which also contributes to the increased price of UHPC.

Recently, researchers have shifted their attention to the development of more economical and less environmentally harmful UHPC mix designs, rather than achieving extremely high compressive strengths (>200 MPa) [10,11,12,13,14,15]. In general, there are three main UHPC cost and carbon footprint reduction strategies: (1) using sand and coarse aggregates from natural gradation processes; (2) minimising the amount of silica fume; and (3) substituting an amount of the high-grade Portland cement with supplementary cementitious materials (SCMs).

Several independent studies [10,11,12,13,15,16] have reported that UHPC cost can be significantly reduced while compromising only 15–20% of its compressive strength by using sand from natural gradation processes with relatively low amounts of silica fume. These findings were adopted in the present study for the development of a cost-effective and efficient UHPC mix. However, the use of SCMs in UHPC production is a much broader research area that covers a wide variety of binders and mix design techniques [7,10]. Although considerable effort has been put into studying the impact of different pozzolanic materials on the performance of UHPC, there are still evident gaps in the research in this field.

The production of high-strength concretes (HPC) often involves the use of pozzolans that promote the formation of calcium silicate hydrates (C-S-H and C-A-S-H). The most common pozzolanic materials used are fly ash (FA) [2,17], silica fume (SF) [18], metakaolin (MK) [19], and milled quartz sand (MQ), which, due to associated health hazards, should be substituted with milled glass (MG) [20,21]. The addition of such mineral admixtures makes concrete denser and reduces the amount of voids, resulting in better resistance to sorption [22].

Research results show that the compressive strength of concrete in which 20% of the cement was replaced with milled window glass decreased during the first days of hydration, and reached the strength of a control composition only after 28–56 days, while at 91 days the compressive strength of the modified concrete even exceeded the strength of the control composition by 5% [23,24,25,26]. According to the literature [27,28,29], SiO_2_ dissolves rapidly, reacts with free portlandite (CH), and behaves like a pozzolan when the sizes of milled glass particles are below 75 μm; the alkali silica reaction stops when the average particle size is approx. 20 μm [30,31]. When coarse ground glass is used as a fine aggregate, only a fraction of the glass particles dissolve, leading to the formation of silica gels in the later stages of hydration. This gel tends to expand, and causes high internal stresses that lead to concrete degradation [32]. Idir et al. [33] observed in their study that glass particles of a size larger than 1 mm caused the formation of an alkali–silica reaction (ASR) gel. On the other hand, smaller glass particles led to the generation of calcium-silicate-hydrate (C-S-H) through pozzolanic reactions. Notably, when the particle size is slightly less than 1 mm, no formation of expansive ASR gel around the particles was observed. Smaller glass particles contribute to improved bonding between the glass particles and the cement paste [34,35]. The replacement of 50% quartz sand with milled glass makes it possible to achieve an exceptionally dense microstructure in the UHPC that is safe from any expansion resulting from ASR. The findings indicate that there is no formation of ASR products at the particle level, and this absence is beneficial for the durability of UHPC, which has extremely low permeability and effectively prevents the entry of alkali into the concrete structure [21].

Typically, MK is used as an additional cementitious material to improve the performance of cement-based materials [34,35,36]. This enhancement is commonly assessed by various parameters such as compressive and flexural strength, water absorption, and drying shrinkage [37]. MK improves the pore structure of cement-based materials by reducing the presence of large detrimental pores and increasing the impermeability of the concrete [38]. Incorporation of MK at cement replacement levels ranging from 0% to 20% produces an observable improvement in compressive strength, splitting tensile strength, modulus of elasticity, and flexural strength of concrete. This enhancement is attributed to the pozzolanic reactivity of metakaolin due to the larger number of OH groups in the crystal structure [39], which contributes to the formation of additional cementitious compounds, thus reinforcing the mechanical properties of concrete. However, a replacement level higher than 20% seems to have an adverse impact on these key properties of concrete [40,41]. Several factors could contribute to this detrimental effect: a lower content of cementitious materials replaced by supplementary additives may have a dilution effect, leading to diminished strength in the concrete; a continuous increase in metakaolin content may trigger excessive pozzolanic reactions, which can alter the balance of the concrete mix, leading to deteriorated mechanical characteristics. The particle size distribution of metakaolin becomes crucial at higher replacement levels. Excess fine particles can induce issues, such as increased water demand or suboptimal particle packing, both of which can compromise the properties of concrete [42].

SF, an SCM containing at least 85% silica and having ultrafine particles, not only improves the strength, but also improves the durability of concrete. This improvement is achieved through reduced porosity and enhanced quality of the transition zones within the concrete [43,44,45]. Additionally, due to its ultrafine nature, SF has a significantly large specific surface area. Consequently, it often requires a substantial dosage of superplasticizer (SP) for effective dispersion of SF particles [46,47]. SF stands out as a prevalent SCM in the production of UHPC. The large surface area of SF particles plays a crucial role not only in filling the pores within the cement matrix, but also in contributing to the cement hydration process [48,49]. However, the currently high cost of SF in many regions hinders significant reductions being made in the initial cost of UHPC. In response, alternative industrial by-products, including, but not limited to, FA, MK [50], and other SCMs, have been proposed as partial substitutes for Portland cement (PC) in UHPC production [51].

In addition to the selection of appropriate raw materials in the design of the concrete mix, it is of utmost importance to achieve a dense microstructure of concrete through an effective particle packing method, both for fine and coarse particles. A dense microstructure is the essential aspect that determines mechanical and durability properties of UHPC [52,53]. Given that SF is approximately 100 times finer than cement [54], there is a considerable size disparity. MK, which is coarser than SF, is initially introduced to fill the gaps between the cement particles. The voids between MK and cement particles are filled with a subsequent addition of SF, which further reduces porosity and improves the overall microstructure of the concrete. Conventionally, SF has been used independently to fill the voids between cement particles with the aim of improving the workability and density, and of improving the performance of mortar or concrete. However, this study suggests that incorporation of SF along with an intermediate-size material such as MK is a more effective approach. The combination of these additives gives more leverage to achieve a successive filling effect, thus optimising the wet packing density and thereby maximising the overall efficiency of the mixture [43,55]. Furthermore, the combined use of MK and SF in concrete can yield a synergistic effect, which proves especially beneficial in the production of high-performance concrete [56,57]. Silicate phases with low hydraulic reactivity found in blast furnace slag are transformed through carbonation treatment into amorphous SiO_2_ and CaCO_3_. These newly formed compounds can then react with portlandite and alumina derived from metakaolin. More specifically, the interaction between the produced CaCO_3_ and metakaolin results in the formation of carbon aluminate phases and stabilises ettringite generated during cement hydration. The consumption of CaCO_3_ further facilitates the pozzolanic reaction of the newly formed amorphous SiO_2_, leading to the production of more C-A-S-H gel. These intricate reactions contribute to the refined microstructure with, ultimately, enhancement of mechanical properties and the impermeability of the material [58].

The aim of this study is to analyse the effect of the following different SCMs: MG, MK, MS, and MQ, and the synergistic effect of the use of a binary pozzolanic additive (MS and MK) on the UHPC structure and properties. The binder replacement rate is kept constant at 10% because, according to the literature, this is the optimal amount of SCM for improving the properties of concrete. The impact of pozzolanic additives is evaluated by examining the hydration kinetics, mechanical and physical properties, and the microstructure of concrete. It is important to compare the properties of UHPC with different pozzolanic additives, especially waste materials, and to create a binary additive that contains waste and can improve the properties of UHPC.

## 2. Materials and Methods

In this study, high-grade Portland cement CEM I 52.5 R (PC) and SCMs such as MQ, MS, MK, and MG were used. The chemical compositions of PC and SCMs are presented in Table 1, and the particle size distribution is presented in Table 2.

As fine aggregate was used, 0/4 mm fraction sand from a local quarry (Lithuania) was used. Chemical admixtures such as superplasticizer based on modified polycarboxylates (SP) and air displacers (ADs) were used.

The compositions of concrete mixtures and the W/B (water/binder ratio) are presented in Table 3. Concrete samples were prepared from 6 different concrete mix compositions: control HPC-1 without pozzolanic additives; HPC-2–HPC-5 with 10% of the cement replaced with different pozzolanic additives, such as MQ, MS, MK and MG; and HPC-6, with a binary pozzolanic additive (5% of MS and 5% of MK). The amounts of other materials in all compositions were the same.

The procedure for the preparation of the samples was as follows: cement, pozzolanic additives, air displacer and sand were dry mixed in a Hobart mixer for 3 min; then, water with a superplasticizer was added, and the mixture was mixed again for 6 min. After mixing, 160 mm × 40 mm × 40 mm samples were moulded. After 24 h, the samples were demoulded and stored in water at a temperature of 20 ± 2 °C for the curing time required for the tests (7 and 28 days). Three samples of each curing age were tested for each property except for compressive strength, for which six samples were tested. These tests were caried out according to EN 196-1 standard [59], but the mixing time, due to superplasticizer efficiency was longer.

The particle size distribution of the materials was analysed using a laser diffraction method (Cilas 1090, 3P Instruments GmbH & Co., Odelzhausen, Germany). Chemical compositions of raw materials (Table 1) were determined using an X-ray fluorescence spectrometer ZSX Primus IV (Rigaku, Tokyo, Japan) equipped with a Rh tube with an anode voltage of 4 kV. Tablet-shaped samples with a diameter of 40 mm were prepared and compressed with a hydraulic press at 200 kN.

The compressive strength was measured using a hydraulic press ALPHA3-3000S (FORM+TEST Seidner&Co. GmbH, Riedlingen, Germany) according to EN 196-1 [59]. The density of the specimens was measured according to EN 1015-10 [60]. The capillary water absorption of the samples was measured according to EN 1015-18 [61] after 28 days of curing, whereas the total water absorption and the degree of structural inhomogeneity were measured according to the methodology described in [62].

The exothermic temperature of concrete mixtures was measured by recording the temperature rise in semi-adiabatic conditions for 36 h. The prepared concrete mixtures (Table 3) were poured into 100 mm × 100 mm × 100 mm moulds, and a thermocouple in a glass pipe was inserted in the middle of each sample to record the temperature increase. Thereafter, the mould was immediately placed in a container insulated with polystyrene foam (thickness 50 mm, thermal conductivity coefficient 0.043 W/(mK)) from inside. The experiments were carried out at (20 ± 1) °C and the temperature was recorded, uninterrupted, until the heat release from the samples considerably decreased. The temperatures of the raw materials and the water used for the concrete mixtures were identical, i.e., (20 ± 1) °C.

The microstructure of the concrete was observed by scanning electron microscopy (SEM) in the secondary electron mode (SE) using a JEOL JSM-7600F device (Tokyo, Japan). Images were obtained from gold-coated surface fractures of the hardened samples by vacuum evaporation technique. The following microscope settings were used: 10 kV and 20 kV voltage; 7–10 mm distance to sample surface.

The XRD analysis was performed using a DRON-7 diffractometer with Cu-Kα (λ = 0.1541837 nm). The following test parameters were used: 30 kV voltage; 12 mA current; and a 2θ diffraction angle range from 4° to 60°, with an increment of 0.02° measured at 0.5 s.

## 3. Results

### 3.1. Temperature Monitoring Results

**Figure 1:**
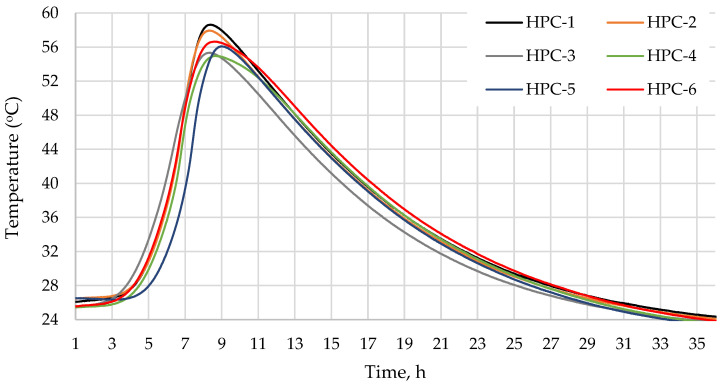
Temperature profile as a function of the hydration time of concrete.

Figure 1 and Table 4 show the temperature profiles of the concrete mixtures during the first 36 h after their preparation. The corresponding sets of the maximum temperature and time to reach it are presented in Table 4. The maximum temperatures of the concrete mixtures are quite similar and range from 55.0 °C (HPC-4 mixture with 10% of MK) to 58.6 °C (mixture HPC-1 without additives), with a difference of approx. 6%. Mixture HPC-2, where 10% of cement was replaced with MQ, showed the smallest temperature variation of 1.7%. Cement hydration was accelerated due to the high specific surface area of the pozzolanic additives MQ and MS, when their particles started to act as hydration centres [63], and probably the highest SiO_2_ content in these additives. Other additives MK and MG slightly retard cement hydration. The maximum temperature was reached the latest in the samples with the MG additive. The hydration time increased 8.3% (HPC-5) because glass particles dissolve more slowly in the alkaline solution and may retard the hydration processes in the early stage. The authors [64,65] found that replacing part of the cement with MG lowered the hydration temperature and reduced the amount of heat released due to the dilution effect, and that the dissolved sodium content was insufficient to accelerate the bonding process or increase the early strength [66]. When the most hydration-accelerating additive MS and hydration-retarding additive MK are used in combination, the maximum temperature is reduced by 2 °C compared to the control sample, whereas the hydration time is similar to that of the MK-containing mixture. Hence, at this stage of strength growth, the dissolving MK coats the MS particles and inhibits the hydration-accelerating effect of MS. Authors [67] reported that low-quality metakaolin retards cement hydration due to the coating of cement grains by MK particles, the formation of ettringite, and the dilution of PC [63].

### 3.2. Physical and Mechanical Properties

**Figure 2:**
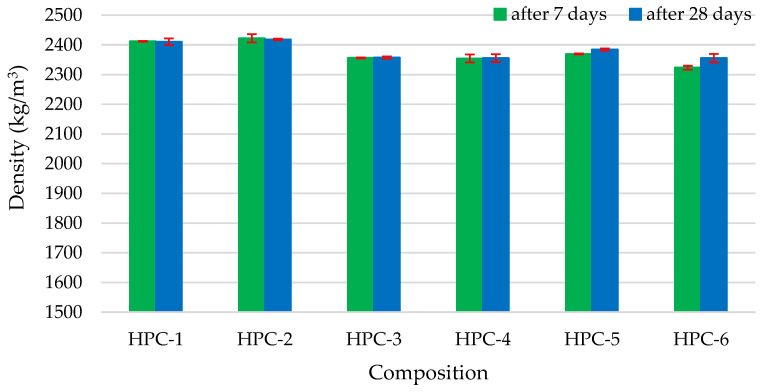
The impact of additive type on density.

Samples HPC-1 and HPC-2 had the highest densities of 2410 kg/m^3^ and 2420 kg/m^3^, respectively, both at 7 and 28 days, whereas the samples of other compositions had a slightly lower density of about 2360 kg/m^3^ (Figure 2). HPC-2 density increased, because MQ is generally inert and engages only in physical interactions during cement hydration. These interactions include the dilution of cement grains, nucleation of cement hydrates, and space-filling; however, MQ exhibits some reactivity at high pH values [68,69,70]. A slight decrease in the density (2%) of other compositions could be caused by the lower density of other pozzolanic additives compared to the density of cement.

**Figure 3:**
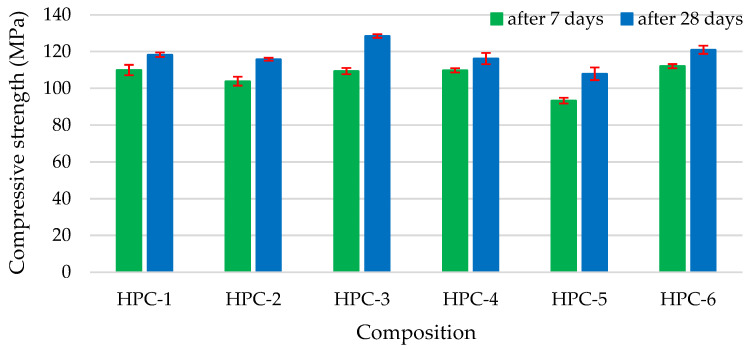
The impact of additive type on compressive strength.

The highest compressive strength (Figure 3) was recorded in sample HPC-3 with 10% of the cement being replaced with MS, and sample HPC-6 containing the binary pozzolanic additive (5% of cement replaced with MS and 5% with MK). At 28 days, the compressive strength of HPC-3 increased 8.6%, and the strength of HPC-6 increased 2.3% although these compositions contained 10% less cement that the control sample. The explanation for such an increase in compressive strength is the large specific surface area and pozzolanic activity of MS particles: a higher conversion of Ca(OH)_2_ into C-S-H results in a denser concrete structure. MK as a pozzolanic material contributes to the enhancement of early-age strength and offers notable improvements in long-term strength. Research [71] indicates that MK modifies the pore structure within cement paste, mortar, and concrete, significantly improving resistance to water transport and diffusion of harmful ions. This enhanced resistance effectively reduces the risk of degradation of the matrix. Compositions with the MG pozzolanic additive had the lowest strength due to the poor bonding of smooth glass particles and the cement matrix [72]. Furthermore, the reduction in strength can be attributed not only to the decreased cement content but also to the retardation of cement hydration. Analysis of SEM results [73] indicates that the agglomeration of ultrafine glass particles contributes to this decline by creating distinct porous zones throughout the material.

### 3.3. Microstructure Analysis

**Figure 4:**
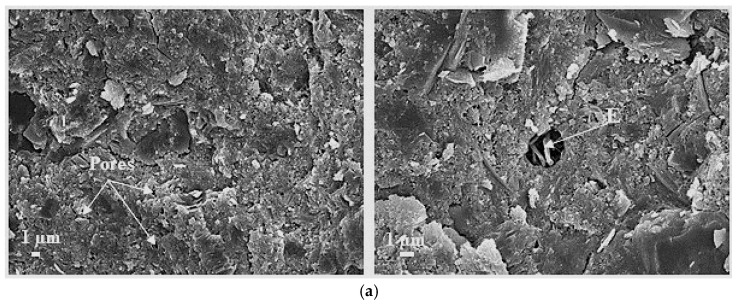

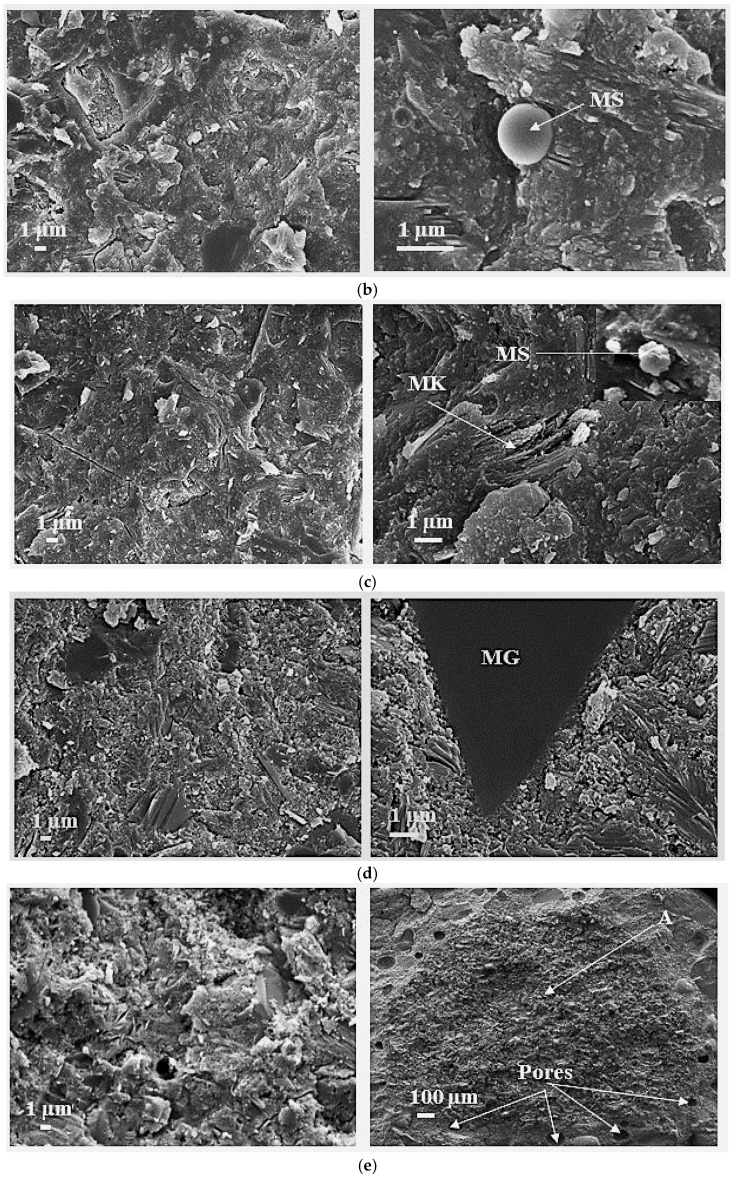

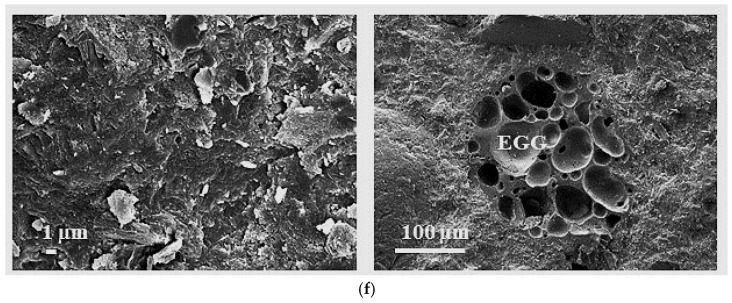
SEM images of HPC concrete: (**a**) HPC-1 (×3000 and ×5000), (**b**) HPC-3 (×3000 and ×20,000), (**c**) HPC-6 (×3000 and ×10,000), (**d**) HPC-5 (×3000 and ×10,000), (**e**) HPC-2 (×3000 and ×25), (**f**) HPC-4 (×3000 and ×250) (E—ettringite; A—fine aggregate; EGG—expanded glass granule).

SEM tests (Figure 4) confirmed the formation of a very dense structure in samples HPC-3 and HPC-6 (Figure 4b and Figure 4c, respectively). Samples of HPC-3 have a very dense structure, MS particles with a very smooth surface are uniformly distributed, and they are well bonded with the cement matrix. In HPC-6 concrete, the surface of MS particles is covered with new hydrates, the growth of which is induced by the reactivity of MK, which bonds well and reacts with the cement matrix. The degree of MK particle reaction was higher in the samples where both additives were used together in comparison with sample HPC-4 modified by metakaolin alone (Figure 4f). The structures of control samples of HPC-1 were slightly more porous, with more pores with ettringite visibly distributed across the entire surface area (Figure 4a). The structures of HPC-5 samples containing MG were more porous and, as mentioned in the paper [68], most of the pores formed near the glass particle (Figure 4d). Concrete samples HPC-2 and HPC-4 had very similar compressive strengths, but their microstructures were different. Samples of HPC-2 revealed the formation of rather large (10–50 µm) air voids around the aggregate grains, whereas no such air voids were observed in other samples. No ettringite was observed in these air voids, which was different from the control sample HPC-1. Samples of HPC-4 with MK additive had a more plate-shaped microstructure. As waste MK from expanded glass granule manufacturing was used in this study, numerous fine glass granules with diameters from 100 to 200 µm, mostly with closed porosity, and which were firmly bonded with the cement matrix were observed in concrete HPC-4 (Figure 4f). These granules absorbed part of water added to the mixture and therefore reduced the porosity of the cement matrix.

### 3.4. Water Absorption and Sorbtivity

**Figure 5:**
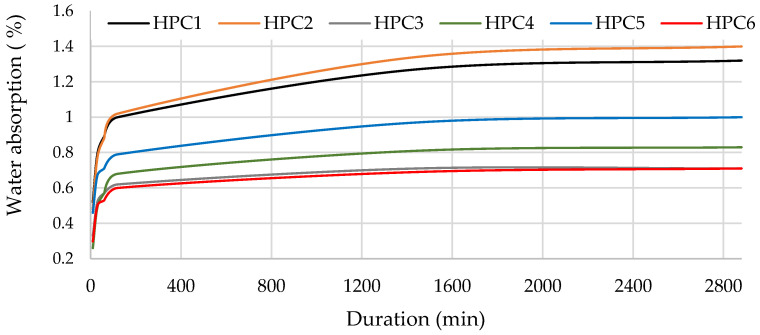
The impact of additive type on water absorption rate.

Concrete samples HPC-3 and HPC-6 with MS had the lowest water absorption rates, as their structures had the highest density. Water absorption of the MS-containing samples reduced by about 50%, from 1.32% to 0.71% (Figure 5), compared to the HPC-1. Traditionally, MS has been used to improve workability, packing density, and the performance of concrete by filling voids between cement particles. However, the proposed approach strategically combines the effects of MK and MS, with the aim of a more comprehensive improvement of the concrete microstructure. MK, which is coarser than MS, is introduced to initially fill the voids between the cement particles. The addition of MS helps to fill the voids between MK and cement particles [52,53,54]. HPC-2 samples containing MQ had the highest water absorption rate, which was 6% higher than the absorption of the control sample. SEM images (Figure 4f) revealed numerous voids with diameters of approx. 50–100 µm around the aggregates in these samples.

**Figure 6:**
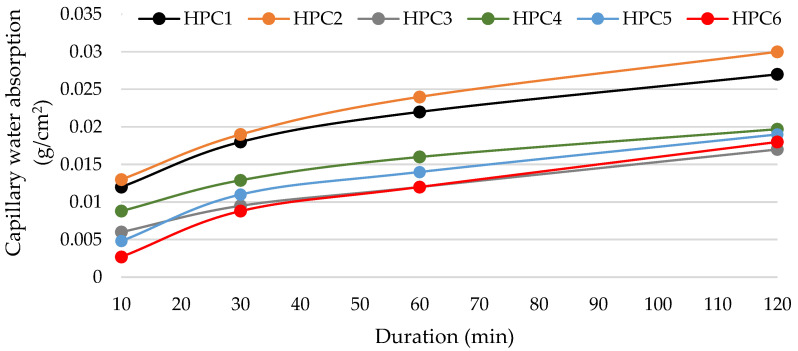
The impact of additive type on capillary water absorption.

**Figure 7:**
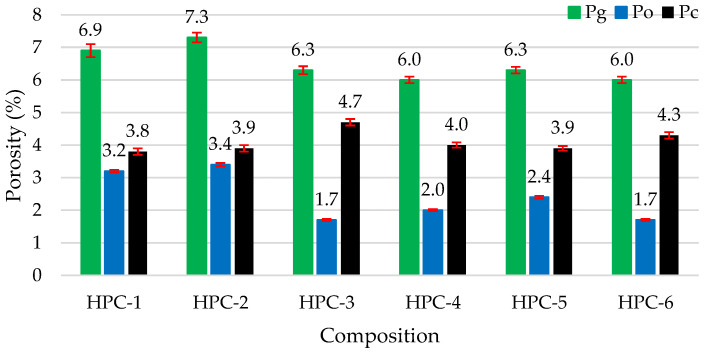
The impact of the additive type on porosity.

The MS-modified samples HPC-3 and HPC-6 had the lowest capillary water absorption (Figure 6), clearly indicating the difference in the number of open capillaries. In 120 min, the capillary water absorption in these samples reduced by about 30% compared to the control sample (HPC-1). This means that MS, either added alone or in combination with metakaolin, has a strong pozzolanic effect, causing faster and more copious formation of C-S-H, which fills, to a certain extent, open pores and capillaries. The highest capillary water absorption was recorded in HPC-2 samples with the addition of milled quartz sand due to the highest open porosity of these samples (Figure 7). It should be noted that HPC-6 samples containing the binary additive had the lowest capillary water absorption for 30 min, and then the absorption rate reached that of HPC-3 samples.

### 3.5. Pore Structure and Resistance to Freeze–Thaw Cycles

The predicted resistance to freeze–thaw cycles was evaluated according to the Kf coefficient calculated applying the methodology described in [74,75]. Briefly, the porosity parameters of concrete were determined by measuring the total, open (Po), and closed (Pc) porosities. These porosity values were then used to predict resistance to freeze–thaw cycles and to calculate the coefficient Kf from the following equation [74,75]:

Kf = Pc/(Po·0.09)(1)

Figure 7 illustrates the results for total porosity (Pg), open porosity (Po), and closed porosity (Pc). Samples HPC-3 and HPC-6 had the lowest open porosity, which, compared to the control sample, decreased by 50%, while the closed porosities increased approximately 24% and 13%, respectively. Samples HPC-6 demonstrated the best total and open porosity results. HPC-4 samples with the addition of metakaolin waste also fit into the lowest total porosity, because metakaolin improves the pore structure of cement-based materials by reducing the appearance of harmful large pores and improving impermeability [38].

**Figure 8:**
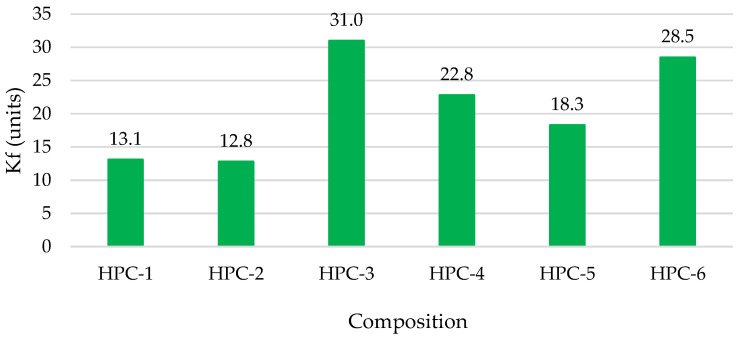
Freeze–thaw resistance prediction coefficient values.

Figure 8 shows the values of the freeze–thaw resistance prediction coefficient calculated from the open and closed porosity values given in Figure 7.

The maximum Kf value used in the prediction method presented in the literature [74,75] only goes up to 12, because conventional concrete is the most frequently studied. The highest Kf values were recorded in HPC-3, HPC-4, and HPC-6 compositions containing MS and MK. The greatest positive change in the Kf coefficient is observed with the increase in closed porosity. MS and MK additives acting independently and, especially, in combination promote cement hydration, and C-S-H and C-A-S-H formation, due to the very small particles of different sizes as well as the pozzolanic activity of these additives. Gradually, the pores are filled to a certain extent by new crystal hydrates. According to other scientific works [76,77], after 3 days of hydration, water penetrates the capillary pores of the initially formed C-S-H and reaches the particle surfaces. During this time, a significant amount of calcium hydroxide is generated as a reaction by-product. The outer hydration products gradually fill the pores between the particles, resulting in a denser structure. Between 3 and 27 days of hydration, the amount of C-S-H increases significantly and a large volume of inner hydration products form within the pores, further enhancing the dense structure of the material. The lowest HPC-2 values could be explained by hydrated products being encapsulated in cement particles covered with MQ, which is not as active as MS and MK. This layer [77] acts as a diffusion barrier, reducing the availability of water and reactants to non-hydrated particles, thereby slowing the overall hydration reaction of the paste, and the hydration products are insufficient to completely fill the voids. This results in the formation of additional voids, contributing to an increase in pore diameter and cumulative pore volume, thereby impacting the microstructural integrity of the material [77]. According to the curve provided in the literature sources referred to above, the concrete tested in ourstudy could resist more than 1000 freeze–thaw cycles; therefore, only the Kf values directly related to the improved freeze–thaw resistance of concrete are given for the comparison of compositions.

The degree of structural inhomogeneity enables the evaluation of the structural inhomogeneity of effective capillaries by their equivalent length, and this is calculated from Equation (2) [62]:

N = (H_max_ − H_min_)/H_min_ (2)

where H_max_ and H_min_ are the rates of the capillary wetting front (after 10 min and 120 min), in mm.

**Figure 9:**
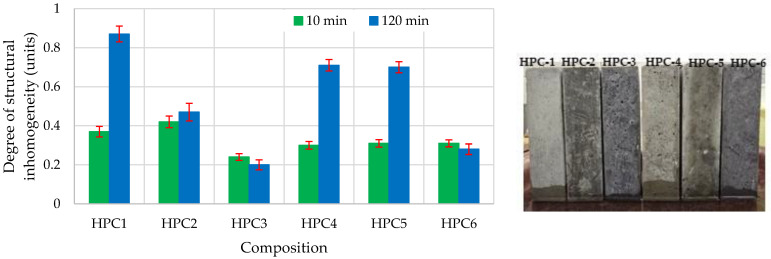
The impact of additive type on the degree of structural inhomogeneity.

**Figure 10:**
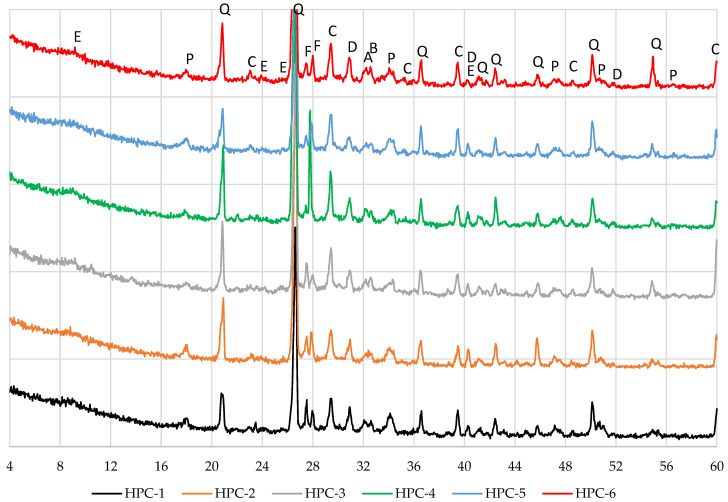
X-ray diffraction patterns of the concrete.

The determined degree of structural inhomogeneity (Figure 9) showed that compositions HPC-3 and HPC-6 had the most homogeneous structures. The density and structural homogeneity of these concrete samples resulted from the most intensive hydration process being used, and the formation of the greatest amount of fine amorphous phases of C-S-H and C-A-S-H, which significantly affects micro- and macro-properties of concrete such as strength and durability [78]. The highest degree of structural inhomogeneity was found in sample HPC-1 due to the ettringite identified in the pores, and it having the highest portlandite content (Figure 10).

### 3.6. Results of X-Ray Analysis

X-ray diffraction analysis of concrete (Figure 10) revealed the same minerals, namely ettringite, quartz, portlandite, calcite, feldspars, dolomite, alite, and belite, existing in all concrete samples irrespective of the pozzolanic additive used.

The lowest portlandite peaks were recorded in samples HPC-3, HPC-4, and HPC-6, whereas sample HPC-1, without pozzolanic additives, had the highest portlandite peak. Compositions with MK showed more intensive calcite peaks. In particular, the interaction between the CaCO_3_ produced and the metakaolin results in the development of carbon aluminate phases, stabilising the ettringite formed during cement hydration [58].

## 4. Conclusions

The effects of pozzolanic additives—milled quartz sand, microsilica, waste metakaolin, milled window glass, and a binary pozzolanic additive (microsilica and waste metakaolin)—on the properties of ultra-high-performance concrete were analysed. The analysis results are summarised below:

- Microsilica, having the highest pozzolanic activity and the largest specific surface area, accelerates hydration the most, whereas milled glass inhibits hydration due to the slower dissolution of the particles and insufficient content of dissolved sodium. Metakaolin, added as a waste product from expanded glass granule manufacturing, also inhibits hydration.
- In spite of the insignificant drop in density (up to 2%), the highest compressive strength was recorded in the samples containing microsilica and the binary additive. At 28 days, the compressive strength of modified samples in comparison with the control sample increased by 8.6% and 2.3% due to the density and the homogeneous structure of concrete. These samples had the lowest total porosity and the highest closed porosity due to the high pozzolanic activity of microsilica. The freeze–thaw resistance prediction coefficient Kf value was also the highest in these compositions. A high Kf value was also observed in the samples with the binary additive and with metakaolin used alone.
- XRD analysis showed that all compositions of concrete contained the same minerals; however, the lowest intensity of portlandite peaks was observed in the samples containing microsilica, metakaolin, and the binary additive metakaolin + microsilica.
- The use of the binary pozzolanic additive is recommended, primarily because metakaolin is a waste product that inhibits the formation of harmful pores in concrete, improves durability, and, above all, reduces the cost of concrete. Microsilica enhances the performance of concrete by accelerating cement hydration and also filling the voids between cement particles.
- This research demonstrates the benefits of using binary pozzolanic additive, which consists of waste metakaolin and microsilica, to optimise the durability of UHPC by analysing the physical and mechanical properties, and the microstructure, of concrete. Research should be continued by replacing a larger amount of cement with SCMs, in order to test the economic and ecological efficiency for the environment, while conducting durability tests under different environmental conditions.

## Figures and Tables

**Table 1:** Chemical composition of PC and SCMs (%).

Material	SiO_2_	CaO	Al_2_O_3_	Fe_2_O_3_	Na_2_O	MgO	SO_3_	K_2_O	TiO_2_	Cl	ZnO	CO_2_	P_2_O_5_
PC	17.0	61.1	4.48	3.17	0.13	2.94	2.05	1.24	0.26	0.02	0.02	7.24	0.09
MQ	99.2	0.07	0.57	0.05	0.03	0.02	0.02	0.02	0.05	–	–	–	–
MS	76.5	0.72	1.20	1.88	1.28	1.36	0.29	2.02	–	0.51	0.08	13.3	0.43
MK	52.4	1.27	39.8	1.03	3.39	0.37	0.08	0.93	0.54	0.01	–	–	0.12
MG	72.3	9.65	1.01	0.17	12.7	3.49	0.28	0.33	0.07	0.02	–	–	–

**Table 2:** Particle size distribution of PC and SCMs.

Material	Average Particle Size (µm)	d_10_ (µm)	d_50_ (µm)	d_90_ (µm)
PC	17.1	0.9	12.6	40.7
MQ	8.5	0.7	5.8	20.5
MS	3.8	0.1	0.7	5.8
MK	20.4	2.6	16.8	44.5
MG	15.1	2.7	13.9	29.2

**Table 3:** Compositions of concrete mixtures, kg/m^3^, and W/B.

Designation	PC	NS	MQ	MS	MK	MG	SP	AD	W	W/B
HPC-1	900	1400					20	2	196	0.22
HPC-2	810	1400	90				20	2	196	0.22
HPC-3	810	1400		90			20	2	196	0.22
HPC-4	810	1400			90		20	2	196	0.22
HPC-5	810	1400				90	20	2	196	0.22
HPC-6	810	1400		45	45		20	2	196	0.22

**Table 4:** Maximum temperature and the time to maximum temperature for each concrete composition.

Parameter\Composition	HPC-1	HPC-2	HPC-3	HPC-4	HPC-5	HPC-6
Max temperature (°C)	58.6	57.9 (−1.7%) *	55.9 (−4.6%) *	55.0 (−6.1%) *	56.4 (−3.8%) *	56.6 (−3.4%) *
Time (h)	8.4	8.3 (−1.2%) *	8.0 (−4.8%) *	8.5 (+1.2%) *	9.1 (+8.3%) *	8.5 (+1.2%) *

* Remark: the numbers in parentheses show the difference from the HPC-1 composition in which the pozzolanic additive was not used.

## Data Availability

All results are presented in the article.

## Abbreviations

The following abbreviations are used in this manuscript:

UHPC	Ultra-high-performance concrete
C-S-H	Calcium silicate hydrates
C-A-S-H	Calcium aluminium silicate hydrate
SCM	Supplementary cementitious materials
MS	Microsilica
MK	Metakaolin
MQ	Milled quartz sand
MG	Milled glass
